# Familiar ethical issues amplified: how members of research ethics committees describe ethical distinctions between disaster and non-disaster research

**DOI:** 10.1186/s12910-017-0203-z

**Published:** 2017-06-28

**Authors:** Catherine M. Tansey, James Anderson, Renaud F. Boulanger, Lisa Eckenwiler, John Pringle, Lisa Schwartz, Matthew Hunt

**Affiliations:** 1 Humanitarian Health Ethics Research Group, McGill University, Montreal, Quebec, Canada and McMaster University, Hamilton, ON Canada; 2 Department of Bioethics, The Hospital for Sick Children, Toronto, ON Canada; 3 University of Toronto, Toronto, ON Canada; 4 McGill University Health Centre, Montreal, Quebec, Canada; 5 Department of Philosophy & Department of Health Administration and Policy, George Mason University, Fairfax, Virginia, USA; 6 Department of Clinical Epidemiology and Biostatistics, McMaster University, Hamilton, ON Canada; 7 School of Physical and Occupational Therapy, McGill University, Montreal, Quebec, Canada; 8 Center for Interdisciplinary Research in Rehabilitation, Montreal, Quebec, Canada

## Abstract

**Background:**

The conduct of research in settings affected by disasters such as hurricanes, floods and earthquakes is challenging, particularly when infrastructures and resources were already limited pre-disaster. However, since post-disaster research is essential to the improvement of the humanitarian response, it is important that adequate research ethics oversight be available.

**Methods:**

We aim to answer the following questions: 1) what do research ethics committee (REC) members who have reviewed research protocols to be conducted following disasters in low- and middle-income countries (LMICs) perceive as the key ethical concerns associated with disaster research?, and 2) in what ways do REC members understand these concerns to be distinct from those arising in research conducted in non-crisis situations? This qualitative study was developed using interpretative description methodology; 15 interviews were conducted with REC members.

**Results:**

Four key ethical issues were identified as presenting distinctive considerations for disaster research to be implemented in LMICs, and were described by participants as familiar research ethics issues that were amplified in these contexts. First, REC members viewed disaster research as having strong social value due to its potential for improving disaster response, but also as requiring a higher level of justification compared to other research settings. Second, they identified vulnerability as an overarching concern for disaster research ethics, and a feature that required careful and critical appraisal when assessing protocols. They noted that research participants’ vulnerabilities frequently change in the aftermath of a disaster and often in unpredictable ways. Third, they identified concerns related to promoting and maintaining safety, confidentiality and data security in insecure or austere environments. Lastly, though REC members endorsed the need and usefulness of community engagement, they noted that there are significant challenges in a disaster setting over and above those typically encountered in global health research to achieve meaningful community engagement.

**Conclusion:**

Disaster research presents distinctive ethical considerations that require attention to ensure that participants are protected. As RECs review disaster research protocols, they should address these concerns and consider how justification, vulnerability, security and confidentially, and community engagement are shaped by the realities of conducting research in a disaster.

## Background

Disasters result in “a serious disruption of the functioning of a community or a society causing widespread human, material, economic or environmental losses which exceed the ability of the affected community or society to cope” [1]. These events occur disproportionately in low- and middle-income countries (LMICs), and are especially devastating in locales where limited infrastructure and surge capacity, combined with high levels of poverty amplify the effects of natural hazards [2]. By disasters, we are referring to catastrophic events (natural hazards) such as earthquakes, floods, hurricanes, tsunamis, and landslides. Such events are commonly described as natural disasters, although we acknowledge that the consequences of natural hazards are shaped by social, political and technological factors [3]. Given the increasing frequency of disasters [4], and the growing number of research protocols that are being implemented during and after disaster events [5], it is important to analyze ethical considerations associated with disaster research, including in which ways disaster research presents distinctive ethical concerns compared to research in non-crisis situations. Doing so can help disaster researchers, as well as research ethics committees (RECs) charged with the review and oversight of disaster research, to better understand and respond to the ethical dimensions of disaster research.

Conducting research during disasters, while often argued to be necessary to improve humanitarian and public health responses to such events [6], can pose multiple logistical and ethical challenges [7]. For example, disasters are never static events, and circumstances and risks change rapidly and often in ways that are difficult to predict. These features of disasters result in challenges for researchers and RECs responsible for reviewing disaster protocols. A range of ethical issues have been identified, such as protocols that are hastily written because of the need to respond quickly after a sudden onset event [8], heightened vulnerability of research participants due to the destabilization and destruction following a disaster [9], the primacy of saving lives following a disaster [10] and cross-cultural issues when international researchers conduct studies during a disaster that has occurred in another country [11].

There is a large set of international and national research ethics guidance documents available^1^ including some texts that are expressly intended for application in disaster and humanitarian contexts [6]. Recently, three contributions providing tailored ethics guidance for disaster and humanitarian research were published with the goal of supporting ethics review. Curry et al. [12] developed an ethics review framework for the Research for Health in Humanitarian Crises (R2HC) grant funding programme. The medical humanitarian organization Médecins Sans Frontières (MSF) (Doctors Without Borders) Ethics Review Board (ERB) has developed a revised version of its ethics review framework to guide the conduct and review of research to be carried out within MSF [13]. In publishing the framework, their intention was to make it available for RECs who might also wish to use it as a guide or structure for their own reviews of humanitarian research protocols [5]. The latest revision of the Council for International Organizations of Medical Sciences’ (CIOMS) International Research Ethics Guidelines for Health-related Research Involving Humans also includes a new section addressing the ethics of research in disasters and outbreaks [14].

While there has been increasing discussion of disaster research ethics in the literature and several useful guidelines/frameworks have been published, the experiences, attitudes and practices of members of RECs that have reviewed disaster research have not been studied. In conducting this research, our goal was to stimulate further discussion about how best to protect participants in disaster research that is conducted in LMICs. Increased understanding of these ethical considerations will help researchers to anticipate the types of concerns that could arise in disaster research. This discussion is also intended to inform REC members who will be involved in disaster research review, especially those who have limited or no experience reviewing such protocols.

## Methods

We undertook a qualitative study using Interpretive Description methodology [15] to investigate REC members’ 1) views regarding ethical concerns arising in natural disaster research in LMICs, and in what ways they understood these concerns to be distinctive when compared to those arising in other research settings, and 2) experiences of reviewing disaster research protocols. Here we present findings related to REC members’ views regarding ethical concerns arising in disaster research. Results related to the experience of reviewing disaster related research, as well as a more extensive description of the methods used to collect and analyze data, are presented elsewhere [16].

Although natural disasters are classified by the Centre for Research on the Epidemiology of Disasters (CRED) as including biological, geophysical, hydrological, meteorological, climatological, and extraterrestrial events [17], we opted not to include research on biological events such as outbreaks of infectious diseases. While there are many overlapping considerations between epidemic research and disaster research, there are also sufficient differences that we felt warranted excluding epidemics. Moreover, there has been more attention in the literature related to research ethics for epidemics [18–20] compared to other disasters.

### Data collection

Members, chairs, advisors and coordinators of RECs were eligible to participate in the study if they had experience reviewing one or more disaster research protocols to be carried out within 2 years of a natural disaster event in an LMIC.

We initiated recruitment of potential interviewees by contacting individuals in our professional networks who we thought might be eligible to participate in the study. We then conducted an extensive Internet search for disaster studies university departments, disaster research centres, non-governmental organizations (NGOs), intergovernmental organizations and other institutions that conduct or support disaster research in LMICs. We sought to identify RECs that were responsible for reviewing research by these organizations. The third step involved reviewing published articles reporting disaster research with the goal of identifying the RECs that had reviewed the research. When an REC was identified through the second or third strategy, a recruitment invitation was sent to the chair of the REC or the most senior individual listed on the REC’s website. To identify additional interviewees, we used snowball sampling; at the end of each interview, we asked the interviewee to suggest other individuals who might be eligible to participate in the research. Six interviewees were recruited through our professional networks, seven were identified via Internet searches, and two interviewees were suggested by other participants (snowball sampling). Emails sent to RECs that we identified through our review of published disaster research did not lead to the recruitment of any participants.

Interviews were conducted from March 2013 to September 2014 in English or French. Fifteen interviewees took part in the study and included individuals living in Australasia, Africa, the Middle East, North America, the Caribbean, and Europe. Interviews lasted from 24 min to 82 min (mean time = 53 min) and were conducted by 2 researchers, both experienced in qualitative research. Collectively the 15 interviewees had experience with 13 RECs involved in the review of disaster research protocols. Details about the interviewees are included in Table 1. (There were 13 unique RECs with 2 RECs represented by 2 interviewees each.)

**Table 1:** Characteristics of interviewees and Research Ethics Committees (RECs)

		Number of interviewees *n* = 15
Gender	Number of women	4/15 (27%)
Type of RECs	Ad hoc committee established during disaster	1
	University	6
	For-profit REC	1
	Other (governmental, international organization, etc.)	7
Location where REC or affiliated organization is situated	High-income countries (HICs)	10
	Low- and middle-income countries (LMICs)	5
Interviewee’s role^a^	REC Chair	5
	REC Member	7
	REC Coordinator or Advisor	4

^a^one had held two roles

Collectively, interviewees had reviewed a wide range of disaster research protocols, although few had reviewed more than two or three. Observational research protocols were more common than interventional studies. Examples of the topics addressed in these studies include: clinical outcomes of amputees after an earthquake, sexual violence in displaced persons’ camps, trials of nutritional supplements for children during/after droughts, incidence of post-traumatic stress disorder and other mental health issues, investigations about tracking of children displaced by disaster, maternal and fetal outcomes of premature labour, and trials of vaccines to prevent infectious disease after a natural disaster. In addition to research on disasters, some interviewees had also reviewed protocols related to research to be conducted during armed conflicts, after industrial accidents and during disease outbreaks. Due to their similarities with disasters, interviewees occasionally discussed research in these other types of crises but were reminded of this study’s focus on disaster research.

Data were collected through semi-structured interviews using open-ended questions. The interview guide was informed by the authors’ previous research on humanitarian health ethics and emergency research ethics [21, 22] and a review of the disaster research literature [23]. Several topics that were identified during the analysis of earlier interviews were incorporated into the interview guide for subsequent interviews with the goal of better understanding these issues or to test emerging insights [15]. While we preferred to conduct interviews in person, due to the wide geographic dispersion of the interviewees we were only able to conduct one face-to-face interview. Thirteen interviews were done using voice-over-internet-protocol (IP) technology, one was conducted in person and one was done by phone. All interviews were digitally recorded and transcribed verbatim. A member of the research team (CT) reviewed each transcript to ensure its accuracy and then wrote a synopsis to capture key points and record her impressions about the interview.

### Data analysis

Interview transcripts were coded and analyzed following the methods of Interpretive Description [15]. Interpretive Description was developed to guide the investigation of complex experiential questions in applied health disciplines. This approach allows for the identification of patterns and commonalities that characterize a phenomenon, while accounting for individual variations, with the goal of creating a coherent account that illuminates a particular domain of experience [15].

We employed an iterative analytic process whereby interviews were coded as soon as the transcripts became available to allow for a responsive relationship between the collection and analysis of data. Coding of transcripts was initiated by CT, using NVivo9 software and by asking broad questions such as “what is going on here?” and “what is this about?” [15] Once coding was complete, similar codes were aggregated into categories that reflected patterns and linkages in the data (MH, CT). These categories were then discussed among team members and broader themes were identified. Transcripts were then reread by two team members (CT, MH) to further refine the analysis and ensure that the provisional analytic structure accurately reflected the experiences and narratives of the interviewees. Thus, through repeated close readings of the transcripts by multiple members of the research team, writing of interview synopses and regular analysis meetings, we developed an in-depth understanding of the data. This enabled us to identify patterns and lines of logical reasoning, consistent with Interpretive Description methodology [15].

We ended recruitment after 15 interviews. By that point, we believed that the analytic structure that we were developing was stable and that additional interviews were unlikely to lead us to revise it further.

### Ethics considerations

The study was approved by the Institutional Review Board of the Faculty of Medicine, McGill University. Each interviewee read, signed and was given a copy of the informed consent form. When an interview was conducted by phone or using IP technology, the consent form was e-mailed to the participant, signed, scanned and returned to the researchers before the interview began. In order to enhance the anonymity of interviewees, they are all referred to with female pronouns in this report.

## Results

A common view amongst all interviewees was that the core principles of ethical research as identified in international research guidelines were relevant and applicable in the context of disaster research. Interviewees did not believe that different ethical principles were necessary for disaster research or that some principles were less important and so ought to be set aside during the ethics review of disaster research. The interviewees did however report that the context of disaster research was distinctive and that this distinctiveness influenced how principles and values ought to be applied in the review of disaster research protocols. We identified four areas where REC members understood ethical principles to be applicable in distinctive ways for disaster research ethics review: 1) assessing the justification for conducting research; 2) addressing vulnerability when it is pervasive; 3) promoting safety, confidentiality and data security in insecure or unstable environments; and 4) gauging the possibility for meaningful community engagement. We present these four themes in the sequential order of a typical REC review. Table 2 summarizes the issues discussed.

**Table 2:** Distinctive ethical considerations for research in disasters identified by REC members

Distinctive issues for disaster research	Considerations for REC review	Examples discussed by interviewees
1. Justification	- Evaluating the social value of disaster research - Considering whether the study must be done in a disaster or if it could be delayed - Analyzing risk/benefit considerations	- Potential to impede disaster response efforts - ‘disaster tourism’ (research which will not generate relevant knowledge or benefit local community)
2. Vulnerability	- Attending to intersecting sources of vulnerability - Avoiding re-traumatization of participants - Assessing expertise of the team - Tailoring consent processes - Responding to changing levels of risk	- Recruiting unaccompanied children, displaced and/or indigent populations
3. Safety, confidentiality and data security	- Promoting participant and researcher safety - Maintaining participant confidentiality - Ensuring data security	- Making contingency plans for evacuation - Using white noise machines - Using mobile technologies to collect and upload anonymized data
4. Community engagement	- Engaging with community before research is developed and throughout its implementation - Identifying additional approaches to seek local input	- Limited time to implement research following sudden onset disaster - Disrupted social systems - Diverse voices and potentially competing interests within communities

### 1) Assessing the justification for conducting research

Interviewees shared the view that research following disasters has significant potential to provide knowledge that could be used to improve future disaster relief activities. They understood disaster research to hold the promise of significant social value as it provides a unique opportunity to produce knowledge and evidence that can help others who experience disasters in the future. One REC member said: *“I disagree with people who think that doing research in a crisis situation is an extreme activity” (participant #11).* She went on to say that not only was it not extreme (i.e., always unacceptably risky or exploitive of participants), disaster research is an important opportunity to develop knowledge about humanitarian efforts. Another interviewee affiliated with the REC of an international organization noted that in the context of a disaster relief operation:

*we seldom step back and say, ‘is what I'm doing, what I'm asking, what we're aspiring to, in any way rational, achievable or justifiable’ … But unless you’re actually measuring and evaluating the impact of your intervention, there’s no way to determine whether you are doing more good than harm (participant #3)*

While emphasizing the crucial role of disaster research, interviewees also argued that not all proposed research projects are ethically acceptable and that RECs ought to consider the justification for conducting a particular project in a disaster setting. For example, though endorsing disaster research as making critical contributions to improving disaster response, an REC member cautioned that research studies should not be implemented in a way that would impede relief efforts:

*so I think the real challenge, and the real task, is to find the way to bring into alignment, the required activities of the disaster responders, and those of the researchers simultaneously (participant #3)*

Another interviewee described some problematic forms of disaster research that were unlikely to yield useful knowledge or provide benefit. She mentioned that after the 2004 Indian Ocean tsunami some researchers:

*… traveled round in countries and described what had happened to people. And I felt that was sort of voyeuristic ... what was to be achieved from this? … and so one of our criteria is ... is the research going to contribute to further knowledge? (participant #6)*

She called this type of project ‘disaster tourism’. Given the potential for some disaster research to be of limited value, while exposing affected communities to inconvenience, burden, or harm, she advised her RECs members to carefully assess the potential social value of research results in their review of disaster research protocols. Another REC member similarly noted that some disaster research protocols she had reviewed were not ethically justified. She stressed that risks and benefits needed to be carefully weighed and that in some instances, research projects that were deemed too burdensome in the initial post-disaster period would be considered acceptable once the acuity of the disaster had abated somewhat. Several interviewees said that if the research question could be answered in non-disaster circumstances, it should not be carried out during or shortly after the crisis.

### 2) Addressing vulnerability when it is pervasive

Heightened and widespread vulnerability following disaster was understood by interviewees to be the primary ethical concern for disaster research review. All of the interviewees recognized that many disaster research participants experience elevated vulnerability, and that disaster often amplifies pre-existing vulnerabilities. One REC chair emphasized the centrality of addressing vulnerability in the review of disaster protocols, stating that: “*I think the vulnerability of the subjects would probably be as important if not [more] important than anything else” (participant #2).* Likewise, an REC advisor saw heightened vulnerability as a clarion feature of disaster research that should be systematically addressed in the ethics review process: “*because if it’s a disaster research [study] then of course vulnerabilities are increased” (participant #12).* On the other hand, several interviewees urged caution when applying the label of ‘vulnerable group’ to all persons affected by a disaster. They noted that the nature and extent of vulnerability varies considerably between and within disaster settings, and amongst groups of research participants. To illustrate this idea, an interviewee described how research participants in disaster settings are diverse, and that researchers and RECs need to acknowledge

*… that there’s different risks for the hurricane survivors … they are likely to be under more stress, more strain, and approach a simple survey much differently than a relief worker who probably is not even from that country (participant #2)*

From this perspective, it is important for RECs and researchers to consider the varying levels and types of vulnerability experienced by disaster research participants.

Interviewees identified several groups or categories of individuals who were particularly susceptible to being harmed in the context of disaster research. For example, an REC chair described that for protocols involving unaccompanied children, her committee provided extremely close scrutiny, saying that they looked at these studies “*through a magnifying glass” (participant #1)* in consideration of the children’s very high levels of vulnerability. Other groups that were identified as especially vulnerable were individuals displaced by a disaster who may not understand local customs or speak the language in the new location, and individuals who were extremely poor. An interviewee described how multiple forms of vulnerabilities can intersect:

*[the potential participants were] very poor, transient in many cases, there were language barriers ... and there were a lot of psychological issues already as a result of just ... their whole life (participant #5)*

Several interviewees described displaced persons as a group that was highly vulnerable because of the legal uncertainty of their situation. An REC member described the ordeals that might have been experienced prior to being enrolled in a research study: “*many of these people have already been through a process of being interrogated, perhaps at the border, perhaps by the police … and then they might be interned to a refugee camp” (participant #11).* Because of such prior experiences*,* participating in research activities such as interviews may be particularly distressing for members of these groups.

A related concern raised by several interviewees was the possibility that certain forms of research could re-traumatize research participants who had just experienced a disaster and its aftermath. This could occur if studies are not designed with careful attention to potential re-traumatization or if studies are implemented too soon after a disaster event. Interviewees emphasized that culturally appropriate psychological support ought to be made available by the research team when study participation may involve psychological stress.

Several interviewees expressed concern that the actions of ill-prepared research teams could cause harm. For this reason, interviewees reported that their RECs considered the research team’s skill and experience in conducting disaster research with vulnerable groups. An REC chair expressed that in her committee:

*issues around vulnerability are very carefully scrutinized. And the appropriateness of a particular researcher to engage in research with certain vulnerable groups might also be a matter of consideration… depending on the sensitivity of the subject (participant #7)*

She linked this concern to the vulnerability of the potential participants. In her experience, novice researchers were less likely to receive approval to conduct research with the most vulnerable groups following a disaster. However, another interviewee noted that there may be a risk for researchers with extensive experience in humanitarian crises to actually become less attentive to vulnerabilities. She described her concern that vulnerabilities become less visible to individuals who are constantly exposed to populations who are at elevated risk of harm: “*where you are dealing with these vulnerable people for the last ten years then they are no longer vulnerable…” (participant #10)*.

The most common approach to protecting vulnerable research participants discussed by the interviewees was seeking their informed consent prior to taking part in a research project. Interviewees acknowledged the limitations of this process especially when participants are traumatized and in precarious situations. They reflected upon whether it was necessary to require written consent, especially in politically fraught situations. Several had occasionally advocated for verbal consent even when researchers had not planned for it in their protocol, particularly when there were concerns about literacy. Verbal consent was also preferred when the only record linking the subject and the research is the consent document, and there were particular security risks for participants in the case of a breach of confidentiality.

Interviewees seldom discussed how changing levels of risk after a disaster should be addressed by RECs and researchers. When specifically asked, several interviewees said that, unless investigators come to them with a concern, the only mechanism available to them for ongoing oversight of evolving risks was the annual review. They described that many RECs require researchers to update the committee annually about the progress of the research and to document any unexpected ethical problems encountered and how these were dealt with. None mentioned an instance where they were contacted by a researcher about an ethical problem encountered between the time of study approval and its annual review. In summary, all of the interviewees spoke of the increased vulnerability of participants after a disaster, and they underlined the need for nuanced consideration of different levels of vulnerability to ensure the provision of relevant protections.

### 3) Promoting safety, confidentiality and data security in insecure or unstable environments

Along with the increased vulnerability of persons who take part in disaster research, avoiding harm and demonstrating respect to participants may also be rendered more challenging. Three commitments of researchers described as possibly more difficult to enact in a disaster setting are promoting participant and research team safety, maintaining participant confidentiality and ensuring data security.

A widespread source of concern following a disaster, and one that was identified by interviewees as affecting both research participants and researchers, is risk of physical harm due to the disaster (such as from aftershocks following an earthquake). As well as risks directly associated with the disaster, there is also an increased risk of violence in the post-disaster period. Participants referred to a number of situations where disasters occurred in locales where political instability was significant, or where instability arose following the disaster. An interviewee reported that her REC approved a study that was later halted due to elevated levels of violence and concerns of physical harm to both researchers and participants. Another interviewee emphasized the need to evaluate the risk of aggression in particularly volatile locations, noting that “*I mean really awful things happened… so yes this is a concern for all research in situations of crisis… in particular in conflicts or violent crisis” (participant #11).* Several of the interviewees felt that, particularly in areas with political instability after a disaster, extra protection for the safety of both research participants and researchers was necessary, including appropriate contingency planning by researchers in anticipation of a potentially deteriorating security situation and a need for evacuation. Research participant security was generally seen as an important consideration for ethics review. However, interviewees had differing experiences regarding the role of the REC in addressing issues related to researcher team security risks. Several participants reported that their REC took risks to researchers’ safety into consideration when assessing the ethical dimensions of a protocol, while other participants described that this fell outside the scope of their review.

The protection of confidentiality was described as a second security-related concern that was regularly discussed in the interviewees’ RECs. One REC member emphasized the importance of protecting data, stating that “*[n]o data is ethically neutral*” (*participant #*3) and researchers have a duty to ensure data security. This concern was seen as especially elevated in disaster settings where there is potential for conflict or violence. For example, an interviewee reported that stolen demographic information could put women, children and elders of a village at risk of violence if it were revealed to armed factions that the men of the village were away. Another interviewee described the importance of safe-guarding the identity of participants as knowledge of their participation could also expose them to harm if the topic was of a sensitive nature e.g., asking displaced persons, especially individuals with precarious legal status in the country, about experiences of oppression or sexual violence.

Several of the interviewees discussed the ways in which different technologies could be used to protect confidentiality. For example, an REC chair reported that her committee asked researchers conducting interviews in a crowded displaced persons’ camp to use a white-noise machine in order to keep discussions from being overheard. Others suggested that tools such as smart phones be used as they allow digital data to be directly collected in a de-identified (anonymous) form, and immediately stored remotely and securely. Data collection via smartphones and tablets was described as being widely accepted in different cultural contexts; one interviewee noted that even when refugees had lost many things, they often still possessed their phones. Other concerns that were raised included the protection from theft of researchers’ computers and phones that contained confidential information, and difficulties related to storing hard copies of documents in a secure manner in the field. An REC member sounded a note of caution, however, emphasizing that there is still uncertainty about how secure research data really are when high tech storage, encryption and transmission devices are used.

### 4) Gauging the possibility for meaningful community engagement

Interviewees identified community engagement as a particular challenge for disaster research.

Though committed to the need and usefulness of community engagement (“a set of practices that help researchers establish and maintain relationships with the stakeholders to a research program” [24]), several interviewees spoke about significant challenges for achieving meaningful community engagement in a disaster setting, especially when protocols needed to be developed and implemented quickly following the disaster. According to one interviewee, “*you don’t know when the disaster is going to hit or where exactly, so it would be hard to set up community approvals and engagement beforehand …” (participant #6)*. Other difficulties described by interviewees for achieving community engagement included the upheaval and strain that is often experienced within local communities after a disaster, as well as the destruction or disruption of key infrastructure such as communication or civil organization. These factors make effective community engagement more difficult than usual.

Interviewees also noted broader issues related to engagement, suggesting that it can be difficult to identify who constitutes “the community” and who are the most appropriate individuals for researchers to consult. Moreover, within communities there may be diverse voices and competing interests. Few strategies were identified for achieving community engagement. However, ways of counterbalancing the difficulty of implementing community engagement in disaster settings were proposed, including collaborating with local researchers and authorities, and partnering with organizations that were working in the region before the event. It was also noted that robust partnership is often not possible unless previous collaborations have taken place, and that some international organizations only accept proposals from local researchers already associated with their organizations. Interviewees acknowledged, however, that these strategies were incomplete solutions to the difficulty of achieving meaningful community engagement.

## Discussion

Based on inductive analysis of interviews with REC members who had reviewed disaster research protocols, we identified four key ethical issues that were seen as presenting in distinctive ways in the context of disaster research conducted in LMICs. Specifically, REC members who participated in our study viewed disaster research as being exceptional in terms of the magnitude and complexity of the ethical issues faced, but not necessarily in terms of their nature. To be responsive to disaster settings, researchers conducting disaster research and RECs who review these studies should pay attention to the distinctive considerations brought to light by this analysis.

Based on our study findings, it appears that the subfield of disaster research ethics does not require a separate set of ethical principles. Our results instead support the view that “there is broad recognition that ethical parameters should and do guide ‘formal’ research” [25] in disaster settings (pg 18). However, several topics would benefit from further analysis to support researchers and RECs to better understand and respond to the particular context of disaster research. We discuss a few of these below.

### Justification and social value

An important consideration identified in this study is related to the justification for research in disasters. Some commentators have argued that there is an ethical imperative to conduct disaster research [26]. Study participants shared this view, yet also expressed the view that research in disasters should only be conducted if it cannot be implemented in a non-disaster setting, and if it cannot be delayed until the acuity of the disaster has decreased. In this sense, the interviewees endorsed the perspective that the justificatory bar for disaster research is higher than for research in more stable circumstances. This perspective is consistent with recent analyses of disaster research ethics [6]. A particular concern discussed by one interviewee was the idea that some research could be a form of “disaster tourism”. She questioned the justification of research that does not have social value and the potential to yield knowledge that could be applied in the current situation or in future disasters. Social value was thus seen as especially salient for judging the appropriateness of conducting a study in a disaster situation.

Several of the interviewees in this study mentioned that concrete plans are needed for the dissemination and implementation of research within the communities where the research is carried out as a means of promoting social value. This is perhaps an area that deserves more attention from RECs. In some instances, knowledge generated may be of crucial relevance to the population immediately affected by the disaster, such as outcomes of vaccine studies. RECs should explore if and how rapid data sharing and dissemination of findings is planned in a given study in order to demonstrate respect for and give back to the communities for their involvement in the research. Likewise, and where applicable, RECs could ask researchers to identify strategies to promote the implementation of successful findings in the participant countries or in future disaster settings. This expectation will vary between settings and may be especially salient for researchers who work with humanitarian organizations. We note, for example, that the ethics review framework of *Médecins Sans Frontières* Ethics Review Board asks researchers to describe plans to “assure access to benefits of the study results if applicable.” pg 9 [13].

### Vulnerability and resilience

Interviewees identified vulnerability as an overriding ethical concern for disaster research, yet vulnerability itself is a contested concept in research ethics, as is the efficacy of research ethics procedures designed to ‘protect’ vulnerable populations [27]. A key challenge in disaster settings is that all individuals living in a locale where a disaster has occurred are likely to experience heightened vulnerability. Indeed, many potential disaster research participants could be considered “doubly” [28] or “especially vulnerable” [10] due to the underlying precariousness of their situation prior to the disaster (e.g., members of a marginalized group) which is then compounded amidst the destruction and upheaval created by a disaster. Such experiences will likely be more widespread when a disaster occurs in a locale of generalized poverty with weak health services and lacking social safety systems. As stressed by Levine et al. [27], the usefulness of ‘vulnerability’ as a concept is reduced when it applies to everyone, or when vulnerability becomes normalized. Given these realities, disaster research ethics will benefit from a conception of vulnerability that can help clarify these considerations and that goes beyond simply categorizing individuals within so-called vulnerable groups. For example, Luna’s [29] idea of ‘layers of vulnerability’ provides a more nuanced way of thinking about this concept, one that may be particularly relevant to disaster research.

In contrast with much of the literature on vulnerability, Luna describes vulnerability as a relational concept that is only applicable to individuals in relation to others, suggesting that an individual is only vulnerable (or not) in a given place, time, circumstance or event. Individuals have more or fewer layers of vulnerability depending on their social context, and their vulnerability increases as additional layers of complexity are added. Our respondents suggested that RECs regularly employ the concept of vulnerability to justify extra scrutiny of disaster protocols and to require special protections to participants recruited for disaster research. This approach is understandable, yet care should be used in attending to the layering of research participant vulnerabilities within disaster-affected populations.

A concept closely related to vulnerability is resilience. According to Zwi et al. “the constant focus on vulnerabilities and problems, and the often almost total lack of recognition of strengths and resilience, can further disempower already exploited groups and individuals. The capacity of refugees and communities in conflict to take an active role in the research process is seldom acknowledged, and undermines the potential for more innovative research which can help generate the evidence for better policy and practice.” [30] The interviewees in this study did not discuss resilience although the concept can also be applied to populations that have experienced a natural disaster. Alongside consideration of vulnerability, the ethics review process may present opportunities for careful consideration of the strengths and resilience of communities who have experienced a disaster and suggest opportunities to engage further with communities and local researchers. In this way, accounting for and building upon community resilience may help address the challenge of community engagement for disaster research, build local capacities for research, and avoid a uni-dimensional view of research participants’ vulnerability.

### Protecting research staff

Interviewees identified security concerns for research staff and research participants in situations of disaster. The interviewees reported different practices and attitudes towards the REC’s responsibility for ensuring researcher safety. Some expressed that the protection of researchers ought to be considered by RECs when they review disaster research. In contrast, several other interviewees reported that, in their institutions, researcher safety was not the responsibility of the REC. These individuals were usually situated in hospitals or universities that have safety committees focused on safety in laboratory and clinical research in their institution. Does the REC have an obligation to evaluate risks to researchers as part of the ethics review? If it is not the role of the REC, how should such risks be attended to and managed? A related consideration, and one discussed by only a few of the interviewees, was responsibilities toward local research staff who may be most exposed to risk (e.g., local surveyors hired to conduct household surveys in insecure settings). While RECs can raise concerns of workplace safety in reviews, they are not equipped or mandated to oversee international labour standards. Researchers nonetheless have an ethical duty to minimize and seek to mitigate risks for the research staff whom they employ. Thus, while it is important that investigators and research staff are protected as they carry out their projects, the logistics of this protection will vary with the context.

### Confidentiality and new technologies

Several of the interviewees identified technological innovations that have the potential to reduce risks in disaster research and protect participant confidentiality, including tools for encryption, anonymization and remote storage. The disaster response sector is an area where many emerging technologies are changing practices and raising new ethical questions [31]. Several of these technologies have the potential for research applications that will also warrant ethical appraisal. For example, the use of drones (or unmanned aerial vehicles (UAVs)) may soon increase in disaster and epidemic research, as it has in disaster relief efforts.^2^ UAVs may be used to transport biological samples, improve mapping of the location of study participant’s residences, and make it feasible to include research subjects previously too remote to participate in research, thereby possibly reducing selection bias or expanding the study population. However, the use of drones may also raise concerns for confidentiality and misuse of data, or biohazard exposure if a drone were to crash or be intercepted. RECs and researchers alike would benefit from further ethical analysis of the issues associated with the use of rapidly changing technologies in disaster settings and disaster research.

### Community engagement

It became clear during our interviews that community engagement is seen as important by REC members, yet it is also acknowledged as a particularly challenging area in the context of disaster research. Multiple features of disaster research render effective community engagement especially difficult. First, disaster research protocols may be developed and implemented rapidly following a disaster and time to engage local communities in project development may not be available. Second, communities that have experienced a disaster may simply have little interest in developing research partnerships given other pressing needs and priorities as they recover from a catastrophic event. Third, compared to non-disaster research, disaster research may be more likely to be undertaken by researchers with limited prior experience in the community, heightening both the need for community engagement and the difficulties associated with carrying it out. Finally, disruption of communication and other infrastructure may also make such engagement more difficult. So while the global health research literature strongly endorses community engagement in all research [24], there have been few suggestions for overcoming challenges to carrying it out in a disaster setting [32]. While acknowledging the challenges identified above, RECs should encourage researchers to identify opportunities to engage communities. One worry is that if community engagement is seen as de facto practically impossible for all disaster research studies, little effort may be placed in seeking opportunities to develop it where and to the extent that it is feasible. For example, in locales with recurrent disasters (e.g., flooding or hurricanes) researchers may have the opportunity to develop closer ties with communities compared to other locales with unpredicted disasters. Researchers can also build connections with local civil society organizations and international NGOs that have longstanding partnerships with communities.

### Limitations

One of the challenges of our study was finding eligible interviewees who were willing to share their experiences with us. We chose to focus on those who had reviewed protocols to be implemented in LMICs and this reduced the pool of eligible participants. In particular, we had difficulty locating eligible individuals living in LMICs. When those approached for participation declined our invitation to participate, the most commonly cited reason was that they did not remember reviewing any disaster protocols. Recruitment challenges likely reflect the lack of collective experience reviewing this type of research globally [25]. We defined disaster research as projects that began collecting data within 2 years of the acute event. Interviewees may have focused more on research projects that were implemented immediately following disasters than on those that were implemented in the months or year after, thus raising issues more relevant to the acute phase of disasters. There might also have been an element of recall bias in our study. Several of the interviewees discussed protocols that they reviewed several years previously, and they had some difficulty remembering specifics about the protocols.

## Conclusion

This article examines ethical considerations associated with disaster research and considers how they are distinctive from those arising in other research settings. The study findings help illuminate, from the perspective of REC members, the ethical dimensions of disaster research. The study suggests a range of issues that researchers should consider in developing research protocols and implementing disaster research, and that RECs should consider in reviewing and monitoring disaster research. These include the justification for conducting research; vulnerability; safety, confidentiality and data security; and community engagement. To stimulate thinking about these issues, researchers and RECs could discuss the questions found in Table 3. These questions are not intended to be a new framework or to be comprehensive, rather they are a sample of the types of questions arising from our analysis that might help draw attention to distinctive ethical considerations associated with disaster research.

**Table 3:** Questions that can draw attention to distinctive ethical considerations of disaster research

Justification
• Will the proposed research increase our knowledge about how to improve humanitarian efforts during a disaster?
• Could this question be answered in a non-disaster setting? • Could the research be delayed and still achieve its scientific objectives?
Vulnerability
• Are the potential participants especially vulnerable because of the disaster? • How do different sources of vulnerability intersect because of the disaster setting? • Does the research team have the requisite experience and skills to carry out the research, including working with vulnerable populations? • How can the risk that the research procedures result in additional harms be minimized (e.g. concern for re-traumatizing participants)?
Safety
• How will study data be secured and the identity of participants kept confidential in a potentially insecure or austere environment?
• Are there safety risks for research participants and research staff particular to this disaster setting, and are there contingency plans in place?
Engagement
• How can community engagement be optimized for this research? • Is it possible to partner with organizations that were already working in this setting prior to the current event?

With expanding disaster research activity, and the growing field of disaster research ethics, researchers and REC members have the opportunity to engage in further discussion and sharing of experiences related to ethics of disaster research. They might also benefit from exploring resources from the field of humanitarian health ethics, such as the repository available at https://humanitarianhealthethics.net/.

There are important differences for research conducted during a disaster compared to research in more ordinary circumstances. However, our findings do not suggest that new research ethics principles are needed for the task of guiding RECs in their deliberations and appraisal of disaster research protocols. Rather, interviewees stressed the need to carefully consider how widely accepted ethical norms for research should be applied to disaster research, and to attend to the ways that familiar ethical concerns might be amplified for research in a disaster.

## Abbreviations

CIOMS: Council for International Organizations of Medical Sciences
CRED: Centre for Research on the Epidemiology of Disasters
ERB: Ethics Review Board
HICs: high-income countries
IP: Internet-protocol
LMICs: Low- and middle-income countries
MSF: Médecins Sans Frontières (Doctors Without Borders)
NGOs: Non-governmental organizations
R2HC: Research for Health in Humanitarian Crises
REC: Research ethics committee
UAV: Unmanned aerial vehicles

## Footnotes

1 Over 1000 guidance documents from 100 countries have been compiled at http://www.hhs.gov/ohrp/international/compilation-human-research-standards/index.html.

2
https://news.vice.com/article/drones-are-being-tested-in-the-fight-against-a-tuberculosis-epidemic-in-papua-new-guinea.
